# The Use of PTI-Marker Genes to Identify Novel Compounds that Establish Induced Resistance in Rice

**DOI:** 10.3390/ijms21010317

**Published:** 2020-01-02

**Authors:** Jonas De Kesel, Ramsés Gómez-Rodríguez, Eli Bonneure, Sven Mangelinckx, Tina Kyndt

**Affiliations:** 1 Department of Biotechnology, Faculty of Bioscience Engineering, Ghent University, Coupure Links 653, 9000 Ghent, Belgium; jonas.dekesel@ugent.be (J.D.K.); ramses.gomezrod@gmail.com (R.G.-R.); 2 Department of Green Chemistry and Technology, Faculty of Bioscience Engineering, Ghent University, Coupure Links 653, 9000 Ghent, Belgium; eli.bonneure@ugent.be (E.B.); Sven.Mangelinckx@ugent.be (S.M.)

**Keywords:** induced resistance, nematode–rice interactions, WGCNA, rice cell suspension cultures

## Abstract

Compounds that establish induced resistance (IR) in plants are promising alternatives for the pesticides that are progressively being banned worldwide. Screening platforms to identify IR-establishing compounds have been developed, but none were specifically designed for monocot plants. Here, we propose the use of an RT-qPCR screening platform, based on conserved immunity marker genes of rice as proxy for IR induction. Central regulators of biotic stress responses of rice were identified with a weighted gene co-expression network analysis (WGCNA), using more than 350 microarray datasets of rice under various sorts of biotic stress. Candidate genes were narrowed down to six immunity marker genes, based on consistent association with pattern-triggered immunity (PTI), both in rice plants as in rice cell suspension cultures (RCSCs). By monitoring the expression of these genes in RCSCs upon treatment with candidate IR-inducing compounds, we showed that our marker genes can predict IR induction in rice. Diproline, a novel IR-establishing compound for monocots that was detected with these marker genes, was shown to induce rice resistance against root-knot nematodes, without fitness costs. Gene expression profiling of the here-described PTI-marker genes can be executed on fully-grown plants or in RCSCs, providing a novel and versatile tool to predict IR induction.

## 1. Introduction

As sessile organisms, plants are highly susceptible for biotic and abiotic stressors. Both can lead to severe yield losses in agricultural crop plants. In the context of a growing world population and increasing urbanization, future gains in food production efficiency will have to come primarily from increased production per acre and not from increases in cultivated area [1,2]. However, increasing yield in an economical and ecological manner will not be evident for staple foods such as rice (*Oryza sativa*). Traditional rice production in flooded paddy fields is threatened by its ecological costs [3], while dry-land rice farming is associated with an increased susceptibility for biotic stress [4,5].

Plants have an innate immune system, represented by Jones and Dangl (2006) as a zigzag model [6]. Pathogen-associated molecular patterns (PAMPs) can be recognized by receptors on the plant cell surface, leading to pattern-triggered immunity (PTI) in the plant [6,7]. PAMPs include compounds such as lipopolysaccharides (LPS) from bacteria, N-acetylglucosamine oligomers ((GlcNAc)_n_) derived from fungi and arthropods [7] and NemaWater (NW), a recently described solution of nematodal components in which yet unknown proteins induce PTI [8]. PTI can be suppressed by pathogenic effectors, which on their turn can be detected intracellularly by plants, ultimately initiating effector-triggered immunity (ETI).

Next to having innate resistance, plants can be triggered to establish a state of systemically enhanced defensive capacity, known as induced resistance (IR) [9,10,11]. Purposely inducing IR as a strategy in disease control has some important advantages over common pesticides [9,10,11,12]. The enhanced defensive status has a broad-spectrum activity that can be maintained for multiple weeks and even several generations. Moreover, IR induction has a limited ecological impact since the plant immune system is boosted, without directly targeting other species such as beneficial insects or microorganisms [10,11,12]. However, establishing IR will most often be outperformed by common pesticides in terms of disease control [13]. Nevertheless, the combination of IR-establishing compounds with pesticides can reduce the amount of pesticides needed, while leading to equivalent—and in some cases even better—results compared to the best pesticide-only treatment [13,14]. Taken together, IR-establishing compounds are expected to become increasingly important as sustainable plant protection strategy [13].

Most IR-inducing compounds have been identified via in planta research where only a limited number of potential candidate compounds were evaluated, often using labor-intensive plant–pathogen interaction experiments. This has led to the identification of compounds such as diproline [15], azelaic acid [16], dehydroascorbic acid (DHA) [17] and riboflavin [18]. A well-known IR-establishing compound that was found via extensive in planta screening is benzo-1,2,3-thiadiazole-7-carbothioic acid S-methyl ester (BTH), the active component of Actigard^®^ [19]. A limited number of in planta screening platforms to screen for IR-inducing compounds have been proposed [20,21,22], but none have been developed for monocot plants, whose immune system shows divergence from those of dicot plants [23].

In order to minimize screening efforts, plant cell suspension cultures (PCSCs) provide simple model systems with notable advantages such as limited needs for special growth infrastructures, homogeneity of the material and fast accessibility of each single cell to exogenous stimuli [24,25,26]. Moreover, PCSCs can be proliferated by subcultivation, leading to a limited need for fresh plant material; this in contrast to screening platforms that use seedlings or plant parts [24,25,26]. Furthermore, the use of undifferentiated, totipotent cells or callus permits to screen for general, tissue-unspecific plant responses. Several in vitro screening platforms have successfully been used to identify compounds that establish IR [27,28,29], but just as for the in planta platforms, none of them were designed for monocots.

IR establishment has been associated with transcriptional reprogramming and the induction of general plant defense responses [30], as well as specific PTI-specific responses [31,32,33]. For example, Ji et al. (2015) demonstrated that upon treatment with the well-known IR-inducing compound β-aminobutyric acid (BABA), rice unveils a PTI-like response, including accumulation of reactive oxygen species, lignin formation and callose deposition [31].

In this study, we hypothesized that transcriptional induction of PTI-marker genes could be used as screening strategy to identify IR-inducing compounds. First, key regulators of the biotic stress response of rice were identified in silico via a weighted gene co-expression network analysis (WGCNA). With this meta-analysis, genes and gene clusters that are consistently associated with certain experimental conditions can be identified [34,35,36]. Similarly, Amrine et al. (2015) distilled a list of 66 hub genes central in the biotic defense response of arabidopsis using a WGCNA [37].

Since the rationale of the hereby-presented study was that induction of PTI genes could be used as a screening strategy for IR-inducing compounds, PTI-responsive genes were selected, via both in planta and in vitro validation. By analyzing a set of well-known and novel IR-establishing compounds for rice, the usefulness of the resulting platform was illustrated. Ultimately, we showed that diproline, a less-described compound detected as IR-inducing by our platform, can effectively and efficiently lower rice susceptibility against the root-knot nematode *Meloidogyne graminicola*.

## 2. Results

### 2.1. In Silico Identification of Rice Genes Associated with Biotic Stress through a WGCNA

Publicly available microarray datasets of rice under compatible interactions with different biotic stressors were downloaded from the ‘GEO DataSets’ database of NCBI [38] (Supplementary Information 1). After quality control, 331 datasets from 22 different studies were retained (Supplementary Information 2). Based on the approximate scale-free topology criterion and the mean connectivity *k*, the power β was set to an optimal value of 6 (Supplementary Information 3). This led to a dendrogram that clustered all rice genes in fifteen different modules (Supplementary Information 3). The expression of four modules was significantly associated with biotic defense, with strongest association for the pink and magenta module (*p* = 2 × 10^−14^ and *p* = 2 × 10^−8^, respectively; Supplementary Information 4). Gene ontology (GO)-enrichment analysis on the joint gene set of these two modules confirmed that 42 of the overrepresented GO-terms were associated with defense responses (Supplementary Information 5).

The here-obtained data were compared with those of Amrine et al. (2015), who studied the biotic stress response of arabidopsis through a WGCNA [37]. For 39 of their 66 hub genes, orthologues were found in our rice network. Almost two-thirds (25/39) of them was assigned to one of the four modules that were significantly associated with biotic defense in rice (Supplementary Information 6).

For all modules, the relationship between gene significance (GS) and module membership (MM), as well as between GS and intramodular connectivity *k* was determined (Supplementary Information 7 and 8, respectively). The GS-value represents the correlation between gene expression and the infection status of the plant, illustrating how robustly the expression of a gene is associated with the biotic defense response of rice. For both relationships, the highest (positive) correlations were observed for the pink and magenta modules. Network parameters and expression levels upon biotic stress, are listed in Supplementary Information 9. Via a parallel WGCNA, studying 217 transcriptome datasets of rice under abiotic stress (Supplementary Information 10), key regulators of the abiotic stress response were also identified (Supplementary Information 11).

Based on the biotic stress WGCNA, 50 candidate genes were selected using four selection criteria: (1) to choose genes that are involved specifically in biotic defense responses, we excluded genes strongly associated with abiotic stress responses, as revealed by the parallel WGCNA described above (Supplementary Information 11); (2) to select for genes consistently upregulated during the course of biotic stress, regardless of the type of pathogen, genes with the highest GS-values were selected from the defense-associated pink and magenta modules; (3) although consistent association with biotic stress was of main interest, genes from the pink and magenta module revealing the highest upregulation during biotic defense were added to the list; (4) to obtain a platform that can identify IR-establishing compounds not only for rice but for monocots in general, only genes having homologues in at least 3 other cereals were considered.

Reverse transcription (RT)-PCR primer pairs were designed for the 50 selected candidate genes (Supplementary Information 12). For 36 genes, these primers were confirmed to be specific by a preliminary RT-qPCR.

### 2.2. In Planta Validation of PTI-Association of Candidate Genes

**Figure 1:**
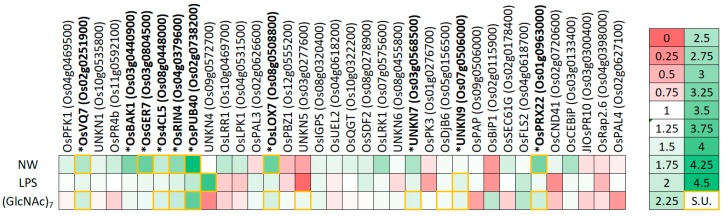
Relative expression levels of the 36 candidate genes in rice plants upon a four-hour treatment with NemaWater (NW; 133 nematode-equivalents/mL), lipopolysaccharides (LPS; 50 µg/mL) and N-acetylglucosamine heptamers (GlcNAc)_7_; 2.5 µg/mL). Each cell color-codes the expression of one of the 36 genes (columns) upon treatment with one of three pathogen-associated molecular patterns (PAMPs; rows). The legend for this color-coding stands right. Yellow-boxed cells indicate significant upregulations (S.U.). Expression levels were determined by RT-qPCR and are expressed relative to mock treated plants. RT-qPCRs were performed with two technical replicates on three biologically independent replicates. Genes in bold and with an asterisk were selected for further analysis.

To select for genes associated with PTI, rice plants were treated with three different PAMPs: NemaWater (NW) [8], lipopolysaccharides (LPS) and N-acetylglucosamine heptamers ((GlcNAc)_7_) [7]. RT-qPCR analysis revealed that after four hours, twelve of the 36 genes were significantly upregulated upon at least one PAMP treatment (Figure 1 and Supplementary Information 13). Based on the consistency of transcriptional upregulation upon all three PAMPs, ten conserved PTI marker genes were selected for further in vitro research (Table 1).

### 2.3. In Vitro Validation of PTI-Association of Candidate Genes

**Figure 2:**
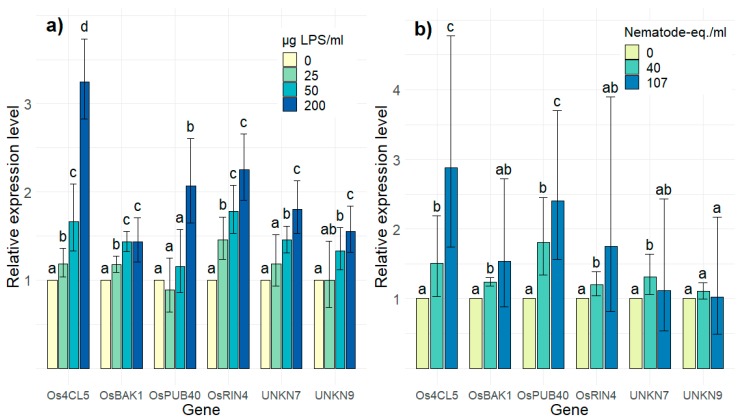
Relative expression levels of the final screening gene set in rice cell suspension cultures (RCSCs) upon a four-hour incubation with (**a**) 25, 50, and 200 µg LPS per mL cells and (**b**) 40 and 107 nematode-equivalents per mL cells. Expression levels were determined by RT-qPCR and are expressed relative to mock treated cells. RT-qPCRs were performed with three technical replicates on two biologically independent replicates. Error bars indicate the 95% confidence interval as calculated by Rest2009 software [40]. Accompanying letters indicate statistical differences, calculated per gene.

The expression of the ten candidate genes (Table 1) was monitored in rice cell suspension cultures (RCSCs) upon incubation with different concentrations of LPS and NW. For four genes (OsGER7, OsLOX7, OsPRX22, and OsVQ7) no, or a highly variable expression, was observed (data not shown). For the other six genes, expression levels are shown in Figure 2a,b upon LPS and NW treatment, respectively (Supplementary Information 14). For both PAMPs, a concentration-dependent induction of gene expression was observed, albeit not always significant for increasing concentrations. These six genes formed the final PTI-marker gene set to screen for IR-inducing compounds.

### 2.4. Induction of PTI-Marker Genes as Proxy to Screen for IR

To investigate the applicability of monitoring the expression of the six selected PTI-marker genes in order to identify IR-inducing compounds, RCSCs were treated with five compounds at concentrations known to establish IR in rice or other plants: diproline (500 µM), Actigard^®^ (250 µM BTH), riboflavin (1 µM), azelaic acid (100 µM), and dehydroascorbic acid (DHA; 20 mM). Diproline, Actigard^®^, riboflavin, and DHA led to a significant upregulation of at least four genes. For azelaic acid, only one gene was upregulated (Table 2 and Supplementary Information 15). Diproline, previously unstudied for rice, induced all six PTI-marker genes.

### 2.5. Diproline as a Novel IR-Inducing Compound for Rice

**Figure 3:**
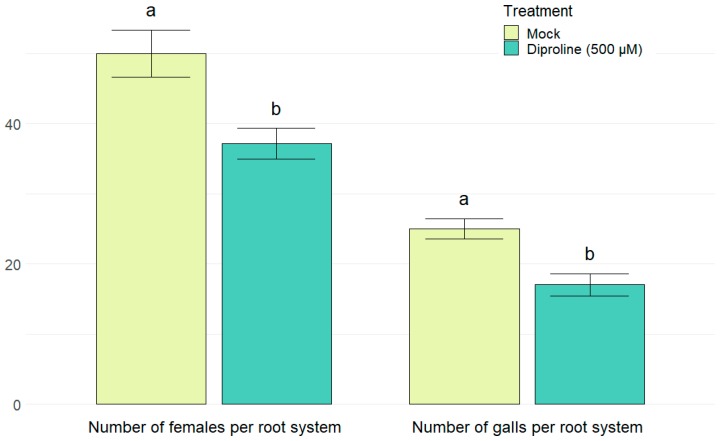
Number of females and galls in root systems of rice plants, 14 days after inoculation with 250 *Meloidogyne graminicola* second stage juveniles per plant. One day before inoculation, fourteen-days-old rice plants were treated with water (mock) or 500 µM diproline. The bars show the average of 24 plants per treatment, analyzed over three replications. To cancel out batch effects, data from the replicate experiments were combined and normalized. Error bars indicate standard errors, while letters represent statistical differences in comparison with the mock treated plants.

**Figure 4:**
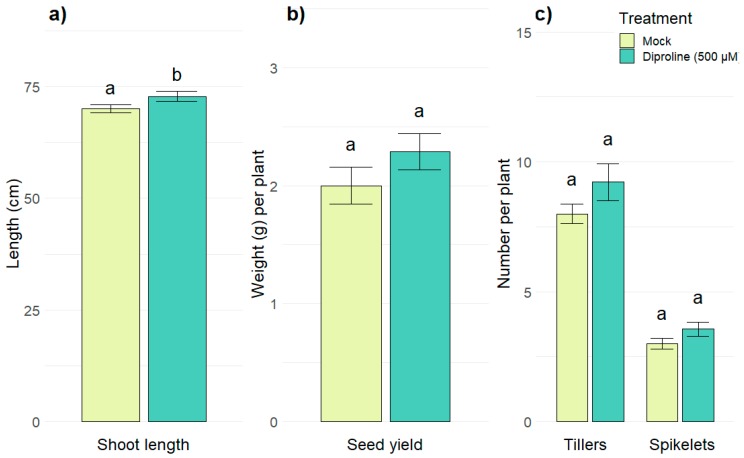
Evaluation of the effects of diproline treatment on plant growth and yield. (**a**) Shoot length, (**b**) seed yield and (**c**) number of tillers and spikelets of four-month-old rice plants after being treated with water or a solution of 500 µM diproline every other week. The bars show the average of 36 plants per treatment, analyzed over two replications. To cancel out batch effects, data from the replicate experiments were combined and normalized. Error bars indicate standard errors. Letters represent statistical differences in comparison with the mock treated plants.

To evaluate the in planta effects of diproline, which significantly induced expression of all six screening genes (Table 2), shoots of rice plants were treated with 500 µM diproline one day before the roots were inoculated with *Meloidogyne graminicola* nematodes. This resulted in a significant reduction of the number of females (*p* = 0.0031) and nematode-induced galls (*p* = 0.0006) 14 days after inoculation (Figure 3 and Supplementary Information 16), illustrating the lowered susceptibility of the rice plants. On the other hand, 100 µM azelaic acid, the least effective treatment according to the PTI-marker gene induction (Table 2), was confirmed to be ineffective in rice plants against nematodes (Supplementary Information 16). As shown in Figure 4, treating rice plants with diproline every other week during their entire lifetime did not affect yield (*p* = 0.1999) nor growth, since the number of tillers (*p* = 0.1351) and the number of spikelets (*p* = 0.1124) were unaffected, while the average shoot length was even significantly longer (*p* = 0.0487; Supplementary Information 16). This indicates the lack of fitness-costs associated with diproline-induced resistance.

## 3. Discussion

To identify compounds that establish induced resistance (IR) in rice, we hypothesized that induction of conserved PTI-responsive genes would be a useful proxy. This hypothesis was based on observations showing PTI-like responses during IR establishment [31,32,33]. Since PTI is the first defense response [6], evaluation of PTI-responsive genes is detectable within hours upon PAMP-treatment [41]. Furthermore, IR-inducing compounds act as extracellular stimuli to induce a defense response, just as PAMPs do [6,7]. The evidence provided in the current manuscript demonstrates that a careful selection of conserved PTI-genes and extensive validation is a valuable strategy to identify a set of marker genes that can adequately detect IR in rice.

The here-described set of marker genes were not only selected on high transcriptional upregulation upon defense activation, but also on the consistency of induction. To assure this consistency of transcriptional upregulation upon various external triggers, whether PAMPs or IR-inducing compounds, we selected genes that were tightly associated with PTI, regardless of the biotic stress evoker or its PAMPs. In order to find genes with such a transcriptional behavior, a weighted gene co-expression network analysis (WGCNA) was combined with a gene expression analysis, both based on >350 publicly available microarray datasets. All genes were eventually selected from the pink and magenta WGCNA modules, which were most clearly associated with the biotic defense response of rice. Furthermore, out of all modules, the pink and the magenta had the most positive correlation between the network parameters ‘gene significance’ (GS) and ‘module membership’ (MM), just as between GS and ‘intramodular connectivity’ (*k*) (Supplementary Information 7 and 8, respectively). This means that central key regulators of the biotic defense response of rice could be chosen unambiguously, since a limited number of genes had the highest values for all three major network parameters.

The potential of WGCNAs to discover conserved, but possibly unstudied defense associated genes becomes strikingly clear when our results are compared with those of Amrine et al. (2015), who studied the biotic stress response in arabidopsis [37]. The orthologous gene pair At2g17220 and Os02g0118200 were in both studies classified in the modules most tightly associated with biotic stress. Moreover, they were among the most interconnected hub genes of these modules. Despite the evidence that this gene could be an elementary plant defense regulator conserved among monocots and dicots, both orthologues have barely been studied. This indicates that the generated WGCNA data might not only be of great value for this research, but also for other studies focusing on fundamental plant defense responses against biotic stressors as well.

Twelve out of the 36 investigated candidate genes were shown to be induced in planta upon at least one PAMP-treatment (Figure 1) and were hence considered as PTI-responsive. The other genes are likely to be ETI associated or they might respond at other time points or in specific plant tissues.

Since large-scale screenings might benefit from working with RCSCs rather than with (parts of) fully-grown plants, the applicability of the PTI-marker genes in RCSCs was also evaluated. For six genes—*Os4CL5*, *OsBAK1*, *OsPUB40*, *OsRIN4*, *UNKN7,* and *UNKN9*—we confirmed a concentration-dependent expression profile upon treatments with LPS and NW (Figure 2). This demonstrates that the hereby-presented gene set can detect PTI-responses in vitro. Notably, *Os4CL5* [42], *OsBAK1* [43] and the arabidopsis orthologue of *OsRIN4* [44] are well known to be involved in plant stress responses, illustrating the relevance of the obtained WGCNA results. On the other hand, *OsPUB40*, *UNKN7* and *UNKN9* are unstudied in rice defense responses, illustrating the discovery potential of this meta-analysis. Not only can the final screening gene set be used to identify novel IR-inducing compounds, it can also be used in the identification and characterization of novel PAMPs. Moreover, unraveling the functions of some newly discovered key PTI regulators, might lead to new insights about the molecular pathways involved in PTI responses.

The applicability of the here-proposed marker gene set to identify IR-inducing compounds for rice was validated by analyzing the effects of well-known IR-establishing compounds and novel candidate molecules. For riboflavin [18] and Actigard^®^ [19], IR-induction was already well described for rice, while IR-induction by DHA has been recently discovered in our lab [17]. For diproline and azelaic acid, IR induction had been described before in dicots, but not in rice or other monocots [15,16]. Table 2 shows that all of the tested IR-inducing compounds led to multiple significant upregulations of the marker gene set in RCSCs, except for azelaic acid. All positive compounds induced at least four out of six genes, making this an appropriate threshold for the selection of promising chemicals. Though mostly significant, the levels of gene induction upon treatments with IR-inducing compounds were minor in terms of fold-change. However, similar induction levels were determined during the transcriptional analysis of the microarray datasets, as well as during the in planta and in vitro validation experiments. Noteworthy, our marker genes were, amongst others, chosen based on strict association with biotic stress and not merely on high differential expression. By considering tight association with defense—determined via the WGCNA—as an important selection criterion, key PTI-regulators were identified, maximizing the chance of induction by IR-establishing compounds with varying mode-of-actions. In conclusion, our gene selection approach led to a robust gene set for the final screening platform, rather than a highly sensitive or specific one.

Diproline, strongly positive according to our in vitro analysis (Table 2), clearly reduced the susceptibility of rice plants against the root-knot nematode *Meloidogyne graminicola* (Figure 3). Because shoots were treated one day before root inoculation, the established IR has a systemic activity. Systemic effects have been described for various IR-establishing compounds [9,10,11], however for diproline these effects were only illustrated in the dicot tobacco [15]. Azelaic acid, which has been shown to be an efficient IR-inducing compounds for dicots [16], was negative according to our analysis in RCSCs (Table 2). Interestingly, azelaic acid also showed no IR-inducing capacity against *M. graminicola* in rice, underlining the robustness of this platform (Supplementary Information 17). This result is consistent with the observations that azelaic acid leads to accumulation of salicylic acid [16], while salicylic acid is a less potent systemic defense inducer in rice against root-knot nematodes when compared to other plant hormones such as jasmonate and ethylene [45].

Since IR-establishment is associated with the induction of a defense response, Martinez-Medina et al. (2016) proposed that—next to efficacy—IR-establishing compounds should also be evaluated in terms of fitness-related costs in treated plants [11]. While for some other IR-inducing compounds, such as β-aminobutyric acid (BABA), growth retardation has been observed [46,47], repetitive treatments with diproline did not affect growth or yield in rice plants (Figure 4). Although the data shown in Figure 3 and Figure 4 clearly validate the efficacy and efficiency of diproline, its molecular mode-of-action is not yet completely known. Wu et al. (2017) demonstrated in tobacco that this compound triggered stomatal closure, induced reactive oxygen species production and stimulated cytosolic calcium ion and nitric oxide production in guard cells [15]. Here we have demonstrated that also in undifferentiated cells PTI-responses are initiated by diproline, illustrating a tissue-unspecific effect. Further investigation to unravel the mode-of-action of diproline in rice is currently being executed in our lab.

Taken together, our results illustrate the validity of the here-presented platform and marker genes to identify novel IR-inducing compounds in rice. To our knowledge, this is the only screening platform in its kind for monocots. Since a main criterion during the selection of candidate genes was the presence of orthologues in other cereals, the here-described screening genes could be easily translatable to studies in other monocot plants. Although in planta validation will always remain essential to evaluate the wanted and possible unwanted effects of positive hits, our marker gene set—applicable both in plant studies as in studies with RCSCs—can be used as valuable prior filtering step before initiating laborious plant infection experiments. Such a prior filtering can make the discovery of IR-establishing compounds more efficient, which is highly desirable in times when many pesticides are getting banned [48] while the efficiency of food production must increase [1,2].

## 4. Materials and Methods

### 4.1. In Silico Identification of Rice Genes Associated with Biotic Stress through WGCNA

#### 4.1.1. Data Acquisition and Pre-Processing

Microarray transcriptome datasets were downloaded from the ‘GEO DataSets’ database of NCBI (www.ncbi.nlm.nih.gov/gds) [38]. ‘Oryza sativa’ was chosen as organism and the ‘Affymetrix Rice Genome Array’ was indicated as the favored platform (GPL2025). In the derived 198 studies, datasets of rice under biotic stress were manually selected. Datasets obtained from genetically modified rice or resistant cultivars were excluded, to study only compatible interactions.

Quality control was done on 352 datasets from 22 studies, using the online ‘Affymetrix QC and pre-processing’ tool (arrayanalysis.org) [49]. Background correction was done via the MAS5 method (‘simpleAffy’ R-package; version 2.48) [50], while subsequent quantile normalization was performed via the RMA method (‘affy’ R-package, version 1.50) [51]. Batch effects from the 22 different studies were removed using the *ComBat* function from the ‘sva’ R-package (version 3.20) [52] and the 20% least varying probes were deleted using the *varFilter* function from the ‘genefilter’ R-package (version 1.54) [53]. As an extra prior filtering step, the function *goodSamplesGenes* from the ‘WGCNA’ R-package was used (version 1.51) [35].

In the end, 331 microarray datasets were withheld to study the general defense response of rice towards biotic stressors, such as bacteria, fungi, arthropods, nematodes, root parasitic plants, and viruses. All studies resulting in these datasets and their quality evaluations are listed in Supplementary Information 1 and 2, respectively.

#### 4.1.2. Network Creation and Clustering

Using the ‘WGCNA’ R-package (version 1.51) [35], the network analysis was performed. With the function *pickSoftThreshold*, the optimal value for the power β was sought. Using this optimal power, automatic network construction and module detection was done with the *blockwiseModules* function. Since it leads to biologically more relevant information [36,54], a signed network was created and the resulting modules whose eigengenes were highly correlated (correlation > 0.75) were merged. During module identification, the more robust ‘biweight midcorrelation’ was used [54].

#### 4.1.3. Identification of Biotic Stress-Associated Modules

After network creation, modules eigengene values (MEs) were used to identify those modules most tightly associated with biotic defense responses [36]. To confirm these associations, a gene ontology (GO)-enrichment analysis was done, using the *g:Profiler* tool (biit.cs.ut.ee/gprofiler/gost) [55]. ‘Biological Processes’ that were significantly overrepresented in the joint datasets of the two modules with the highest MEs were identified as such. ‘Ordered query’ was selected as analysis option, since input genes were ordered along GSs. To compare our data with the 66 hub genes of the arabidopsis WGCNA of Amrine et al. (2015) [37], the 66 rice orthologues were queried using rice.plantbiology.msu.edu [56].

#### 4.1.4. WGCNA on Transcriptome Datasets of Rice under Abiotic Stress

In a similar way as described in Section 4.1.1, Section 4.1.2 and Section 4.1.3, transcriptome datasets of rice under abiotic stress were used to build an abiotic stress response network. After quality control, 217 transcriptome datasets were withheld, comprising cold stress, stress because of pollutants, drought stress, salt stress, anoxia, water stress and nutrient starvation. All used datasets for the abiotic WGCNA are listed in Supplementary Information 10 and the resulting network parameters in Supplementary Information 11.

### 4.2. In Planta Validation of PTI-Association of Candidate Genes

#### 4.2.1. Gene Selection

Based on the WGCNA results, 100 candidate genes were selected for further investigation. These genes were chosen out of the two modules from the biotic network that were most strictly associated with defense responses. For each of the two modules, 25 central key regulators were selected. Upon confirmation of positive correlations between the network parameters ‘gene significance’ (GS) and ‘intramodular connectivity’ (*k*), as well as between GS and ‘module membership’ (MM) (shown in Supplementary Information 7 and 8, respectively), 50 central key regulators could be chosen based on only one network parameter, being gene significance (GS). They were supplemented with genes that had the highest differential expression upon biotic stress, based on the analysis of the microarray datasets. Again, 25 genes per module were selected based on this criterion. To analyze the latter, the R-package ‘RankProd’ (version 2.44) was used [57].

After removing duplicates, the remaining genes were filtered based on two additional criteria. First, central regulators of abiotic stress responses, found via the constructed abiotic stress response network (see Section 4.1.4), were removed. More specifically, candidate genes that were among the top 5% of the abiotic network in terms of GS, or in terms of transcriptional upregulation, were excluded. Secondly, genes were only withheld if a homologue could be found via the ‘Orthologues’ tab on plants.ensembl.org [58] in at least three of the following cereals: wheat, barley, maize and sorghum. As homology criterion, a sequence identity of >30% was set [59]. For the 50 resulting genes, specific RT-qPCR primer pairs were designed.

#### 4.2.2. In Planta Evaluation of Gene Expression Profiles upon PTI Induction

Seeds of *Oryza sativa* ssp. *japonica* cv. Nipponbare (USDA; Washington D.C. USA,) were germinated for three days at 30 °C on wet paper cloths. Seedlings were transferred into a mixture of quartz sand (Sibelco; Antwerp, Belgium) with water absorbent polymer and grown at 28 °C under a 16 h/8 h light/dark regime, as described by Reversat et al. (1999) [60]. To evoke PTI responses, rice plants were treated with three different PAMPs. Lipopolysaccharides (LPS) from *Pseudomonas aeruginosa 10* (Sigma-Aldrich; USA, Missouri, Saint Louis) was used in a concentration of 50 µg/mL as bacterial PAMP [7]. N-acetylglucosamine heptamers ((GlcNAc)_7_; available as Hepta-N-acetyl chitoheptaose at Isosep; Tullinge, Sweden), an arthropodal and fungal PAMP, was used in a concentration of 2.5 µg/mL [7]. NemaWater (NW) was used as nematodal PAMP [8]. After extraction of 40000 *Meloidogyne graminicola* J2s through a 200 µm filter for 2 days at 30 °C in tap water, the nematodes were sedimented and the upper water layer was removed. The lower 300 mL, containing the nematodes, was shaken overnight at 100 rpm. To remove the nematodes the next day, they were sedimented and the upper 100 mL was filter sterilized and used as NW. This led to NW with a concentration of 133 “nematode-equivalents per mL”. All PAMP solutions were prepared in distilled water and contained 0.02% (*v*/*v*) Tween20 (Duchefa Biochemie; Haarlem, The Netherlands). Water with Tween20 was sprayed on the control plants as mock treatment. Plants were sprayed with vaporizers with a fine mist until run off.

Per treatment, three biological replicates were used, each consisting of five plants. After four hours, the shoots were collected and snap-frozen in liquid nitrogen. RNA-extraction was done using the RNeasy Plant Mini Kit (Qiagen; Hilden, Germany,) and DNase-treatment was executed with DNase I (ThermoFisher scientific; USA, Massachusetts, Waltham). Three micrograms of RNA was used for cDNA synthesis, using the Tetro cDNA Synthesis Kit (Bioline; London, UK). RT-qPCRs were performed with two technical replicates, using a CFX Connect Real-Time PCR Detection System (BIO-RAD; Hercules, California, US). The SensiMix SYBR HI-ROX kit (Bioline; London, UK) was used. Following protocol was used for RT-qPCRs: 10 min at 95 °C, 40 cycles of 25 s at 95 °C, 25 s at 58 °C and 20 s at 72 °C. Finally, a melting curve analysis was performed. The sequences of all used primers are listed in Supplementary Information 12.

#### 4.2.3. Data Analysis

To ascertain stable expression of the reference genes and to control variability of the two technical replicates, qBase+ [61] was used. Using REST2009 [40], the expression levels upon PAMP treatments were investigated in comparison with mock treated plants. Gene expressions that had 95% confidence intervals completely exceeding or completely below the reference value of 1, were considered as significantly upregulated or downregulated, respectively.

### 4.3. In Vitro Validation of PTI-Association of Candidate Genes

#### 4.3.1. Establishment of Rice Cell Suspension Cultures (RCSCs)

To establish RCSCs, rice seeds were sterilized for 5 and 30 min with 75% ethanol and a 5% HAZ TABS solution (novolab; Geraardsbergen, Belgium), respectively. After rinsing with sterile water, seeds were cultivated for 30 days on callus inducing medium (Supplementary Information 18). The obtained callus pieces were then proliferated further in liquid amino acid (AA) medium (Supplementary Information 18) using disposable CELLSTAR cell culture flasks (greiner bio-one; Vienna, Austria). Every five to seven days, cells were subcultivated by decanting AA medium and replacing it with fresh medium.

#### 4.3.2. Cell Treatments and RT-qPCR

Four days after subcultivation, cells from multiple RCSCs were separated from the callus clumps using a 70 µm falcon cell strainer (VWR; Pennsylvania, Radnor, USA) and pooled together. For each treatment, 40 mL cells were transferred to new CELLSTAR cell culture flasks (greiner bio-one; Vienna, Austria) and treated with 0.4 mL 100x LPS solutions, to obtain final concentrations of 200, 50, and 25 µg LPS per mL AA medium. For a parallel experiment, NemaWater (NW) was made as described in Section 4.2.2. In summary, 12000 and 9000 nematodes were shaken overnight in 6.0 and 4.5 mL tap water, respectively. 1.0 and 0.8 mL of the aforementioned solutions were filter sterilized and used as NW to treat 50 and 15 mL cells, respectively, leading to a concentration of 40 and 107 nematode-equivalents per mL cells. Fresh AA medium was used as mock treatment. After four hours, cells were transferred to 50 mL tubes (Novolab; Geraardsbergen Belgium), centrifuged for five minutes at full speed and after the removal of the supernatant snap-frozen in liquid nitrogen.

RNA-extraction was done using TRI Reagent (Sigma; Saint Louis, Missouri USA), in combination with Direct-zol RNA purification columns (Zymo Research; Irvine, California, USA,). DNase-treatment was executed with DNase I (ThermoFisher scientific; Massachusetts, Waltham, USA) and cDNA synthesis was done using the Maxima First Strand cDNA Synthesis Kit for RT-qPCR (ThermoFisher; Massachusetts, Waltham, USA). The sequences of all used primers are listed in Supplementary Information 12. RT-qPCRs were performed with three technical replicates on two biologically independent replicates. Further details about the execution of the RT-qPCRs and the statistical analysis can be found in Section 4.2.2 and Section 4.2.3, respectively.

### 4.4. Induction of PTI-Marker Genes as Proxy to Screen for IR

RCSCs were treated for four hours with different compounds: 500 µM diproline, 250 µM S-methyl 1,2,3-benzothiadiazole-7-carbothioate (BTH) formulated as Actigard^®^ 50WG (Syngenta; Basel, Switzerland), 1 µM riboflavin (Alfa Aesar; Massachusetts, Ward Hill, USA), 100 µM azelaic acid (Sigma-Aldrich; Missouri, Saint Louis, USA) and 20 mM dehydroascorbic acid (DHA - Sigma-Aldrich; Missouri, Saint Louis, USA). Per sample, 40 mL of cells were treated with 20× solutions. All other downstream steps were done as described in Section 4.3.2. Diproline (*Cyclo*(l-Pro-l-Pro)) was synthesized starting from H-Pro-OMe·HCl (Apollo Scientific; Bredbury, UK) and Boc-Pro-OH (Sigma-Aldrich; Missouri, Saint Louis, USA) with a procedure modified from Campbell et al. (2009) [62]. DMF was used as solvent, OxymaPure (ChemPUR; Karlsruhe, Germany) was added as additive and the final compound was purified with a preparative HPLC. The equipment used was an Agilent 1100 Series system (Agilent; Santa Clara, California, USA) with a Supelco Ascentis C18 column (21.2 mm × 150 mm, 5 µm) (Sigma-Aldrich; Missouri, Saint Louis, USA).

### 4.5. Nematode Infection Experiments

Rice (*Oryza sativa* ssp. *japonica* cv. Nipponbare—USDA; Washington D.C., USA) plants were grown for two weeks as described in Section 4.2.2. After fourteen days, 500 µM diproline or 100 µM azelaic acid containing 0.02% (*v*/*v*) Tween20 (Duchefa Biochemie; Haarlem, The Netherlands) was sprayed on the shoots untill run off. Water with Tween20 was sprayed on the control plants as mock treatment. One day later, each root system was inoculated with 250 *Meloidogyne graminicola* nematodes. The infection level of the plants was evaluated two weeks after inoculation. To visualize galls and nematodes, root systems were boiled for three minutes in a 12.5% raspberry red solution. Afterwards, roots were washed with running tap water and then destained in acid glycerol. To evaluate the infection levels of the plants, the number of females and the number of galls was counted per root system. The infection experiment was repeated one and three times, respectively for azelaic acid and diproline. Per repetition, each treatment consisted out of eight individual plants. The resulting data from the three repetitions were combined and normalized, so that the control plants had on average 50 females and 25 galls per root systems. Statistical differences were determined with a heteroscedastic two-sided *t*-test.

### 4.6. Long-Term Effects of Diproline Treatments on Plant Growth and Development

Rice (*Oryza sativa* ssp. *japonica* cv. Kitaake) plants were grown for four months in a greenhouse at 32 °C under a 12 h/12 h light/dark regime. Every two weeks, plants were treated with 5 mL of a 500 µM diproline solution containing 0.02% (*v*/*v*) Tween20 (Duchefa Biochemie; Haarlem, The Netherlands). Water with Tween20 was sprayed on the control plants as mock treatment. After four months, shoot lengths, numbers of spikelets, numbers of tillers, and seed yields were quantified per plant. This experiment was repeated twice, with 24 and 12 plants per treatment, respectively. The resulting data from the two repetitions were combined and normalized, so that the control plants were on average 70 cm long, had respectively eight and three tillers and spikelets and resulted in a seed yield of two gram per plant. Statistical differences were determined with a heteroscedastic two-sided *t*-test.

## Figures and Tables

**Table 1:** The ten candidate pattern-triggered immunity (PTI)-marker genes selected for further in vitro investigation, with their Rap-DB annotations [39].

Gene	Name	Annotation
Os08g0448000	*Os4CL5*	4-coumarate–CoA ligase 5
Os03g0440900	*OsBAK1*	BRASSINOSTEROID INSENSITIVE 1-associated receptor kinase 1
Os03g0804500	*OsGER7*	Germin-like protein 3-7
Os08g0508800	*OsLOX7*	Lipoxygenase 7, chloroplastic
Os01g0963000	*OsPRX22*	Similar to Peroxidase BP 1 precursor
Os02g0738200	*OsPUB40*	Zinc finger, RING/FYVE/PHD-type domain containing protein
Os04g0379600	*OsRIN4*	Rin4 domain containing protein
Os02g0251900	*OsVQ7*	Similar to Tobacco rattle virus-induced protein variant 2
Os03g0568500	*UNKN7*	Uncharacterized protein family UPF0136
Os07g0506000	*UNKN9*	Protein of unknown function DUF300 family protein

**Table 2:** Relative expression levels of the screening gene set upon four-hour incubations with five IR-inducing compounds (columns 2–6). Expression levels were determined by RT-qPCR and are expressed relative to mock treated cells. RT-qPCRs were performed with three technical replicates on two biologically independent replicates. As comparison, the relative expression values detected in vitro and in planta upon PAMP treatments are listed in columns 7–8 and 9–11, respectively (shown in Figure 1 and Figure 2, respectively). The upregulations determined in the WGCNA are displayed in column 12. For columns 7–8, the data correspond with the 200 µg LPS/mL and 107 nematode-equivalents/mL treatments, respectively. Grey cells with bold data represent significant differential regulations.

1	2	3	4	5	6	7	8	9	10	11	12
	*In Vitro*							*In Planta*			*In Silico*
	Dipro-line	Acti-gard^®^	Ribo-flavin	Azelaic acid	DHA	LPS	NW	LPS	NW	(Glc-NAc)_7_	
*Os4CL5*	**1.860**	1.664	**1.809**	1.075	**2.166**	**3.249**	**2.878**	1.359	**2.172**	**1.720**	**1.774**
*OsBAK1*	**1.329**	1.434	**1.454**	**1.288**	**1.688**	**1.434**	1.532	1.200	3.270	1.334	**1.208**
*OsPUB40*	**1.479**	**1.157**	1.338	1.102	**5.918**	**2.071**	**2.403**	2.203	**4.539**	**3.687**	**1.853**
*OsRIN4*	**1.602**	**1.778**	**1.516**	1.053	**0.34**	**2.250**	1.753	1.627	**3.051**	**2.098**	**1.401**
*UNKN7*	**1.288**	**1.454**	**1.094**	1.106	**1.821**	**1.803**	1.117	1.525	2.507	**1.671**	**1.244**
*UNKN9*	**1.253**	**1.333**	**1.261**	1.121	0.809	**1.553**	1.021	**1.579**	1.183	**1.520**	**1.129**

## Supplementary Materials

Supplementary Materials can be found at https://www.mdpi.com/1422-0067/21/1/317/s1.

## Abbreviations

IR	Induced resistance
RCSC	Rice cell suspension cultures
WGCNA	Weighted gene co-expression network analysis
PTI	Pattern-triggered immunity
PAMP	Pathogen-associated molecular pattern
NW	NemaWater
ETI	Effector triggered immunity
DHA	Dehydroascorbic acid
BTH	Benzo-1,2,3-thiadiazole-7-carbothioic acid S-methyl ester
PCSC	Plant cell suspension culture
BABA	β-aminobutyric acid
GO	Gene ontology
GS	Gene significance
MM	Module membership
k	Intramodular connectivity
ME	Module eigengenes
RT-qPCR	Revere transcription-quantitative polymerase chain reaction
LPS	Lipopolysaccharides
(GlcNAc)_7_	N-acetylglucosamine heptamer
