# Supplementary material for: The Use of PTI-Marker Genes to Identify Novel Compounds that Establish Induced Resistance in Rice
Source: Int J Mol Sci. 2020 Jan 2;21(1):317. doi: 10.3390/ijms21010317 (PMC6981679; doi:10.3390/ijms21010317)
Supplement: Supplementary file 1 [file ijms-21-00317-s001.zip › Supplementary_Information_3-selection power beta and cluster dendrogram.docx]

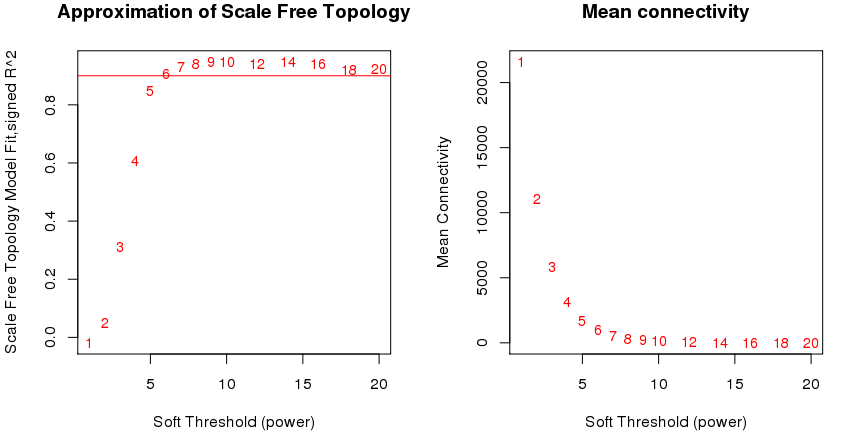


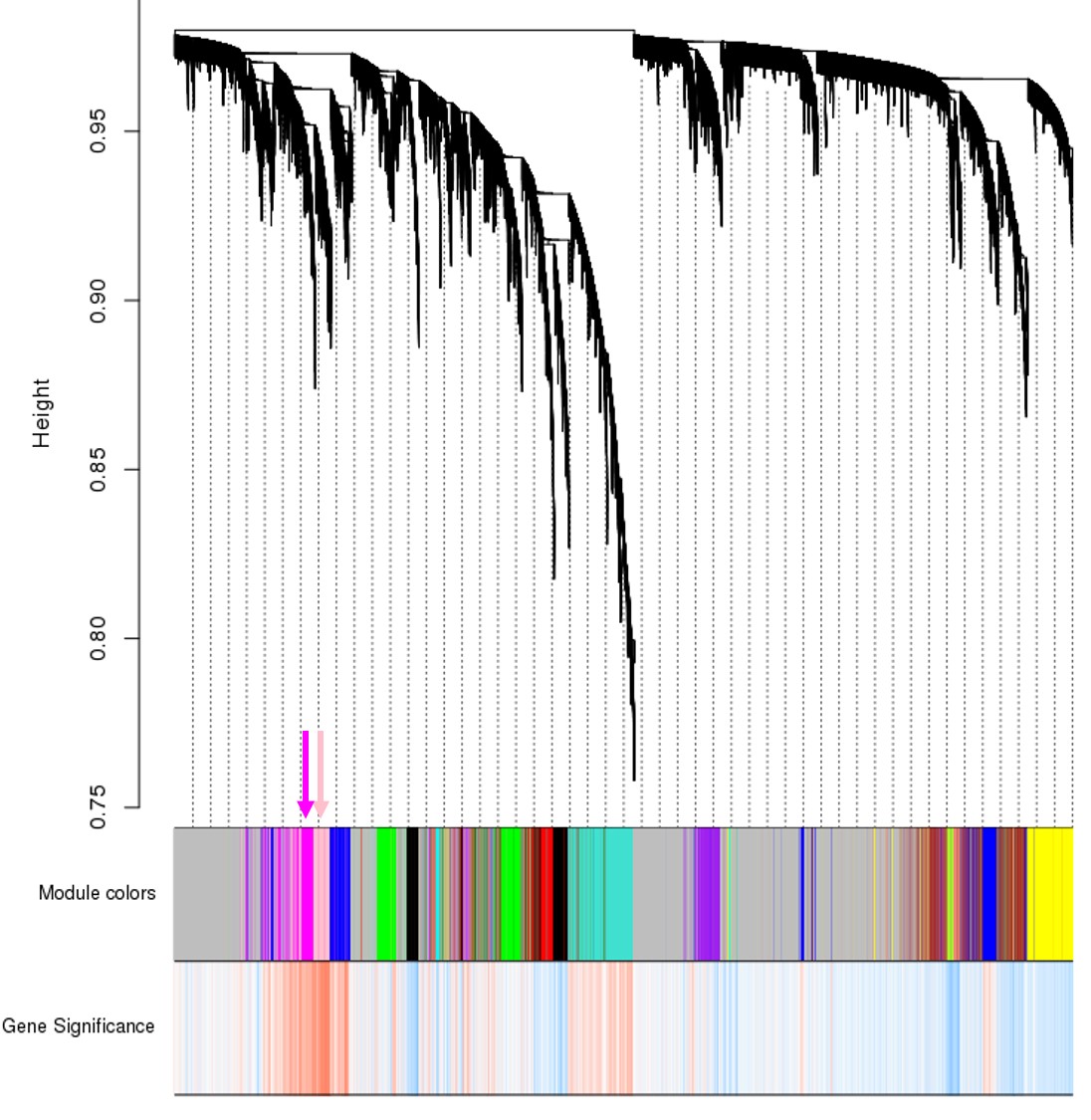


**Supplementary Information 3:** Above: relation between the R² value for the scale free topology model fit & power β (left) and relation between mean connectivity *k* & power β (right), for the biotic WGCNA network. Below: the resulting cluster dendrogram.
