# Supplementary material for: The Use of PTI-Marker Genes to Identify Novel Compounds that Establish Induced Resistance in Rice
Source: Int J Mol Sci. 2020 Jan 2;21(1):317. doi: 10.3390/ijms21010317 (PMC6981679; doi:10.3390/ijms21010317)
Supplement: Supplementary file 1 [file ijms-21-00317-s001.zip › Supplementary_Information_8-GS vs k.docx]

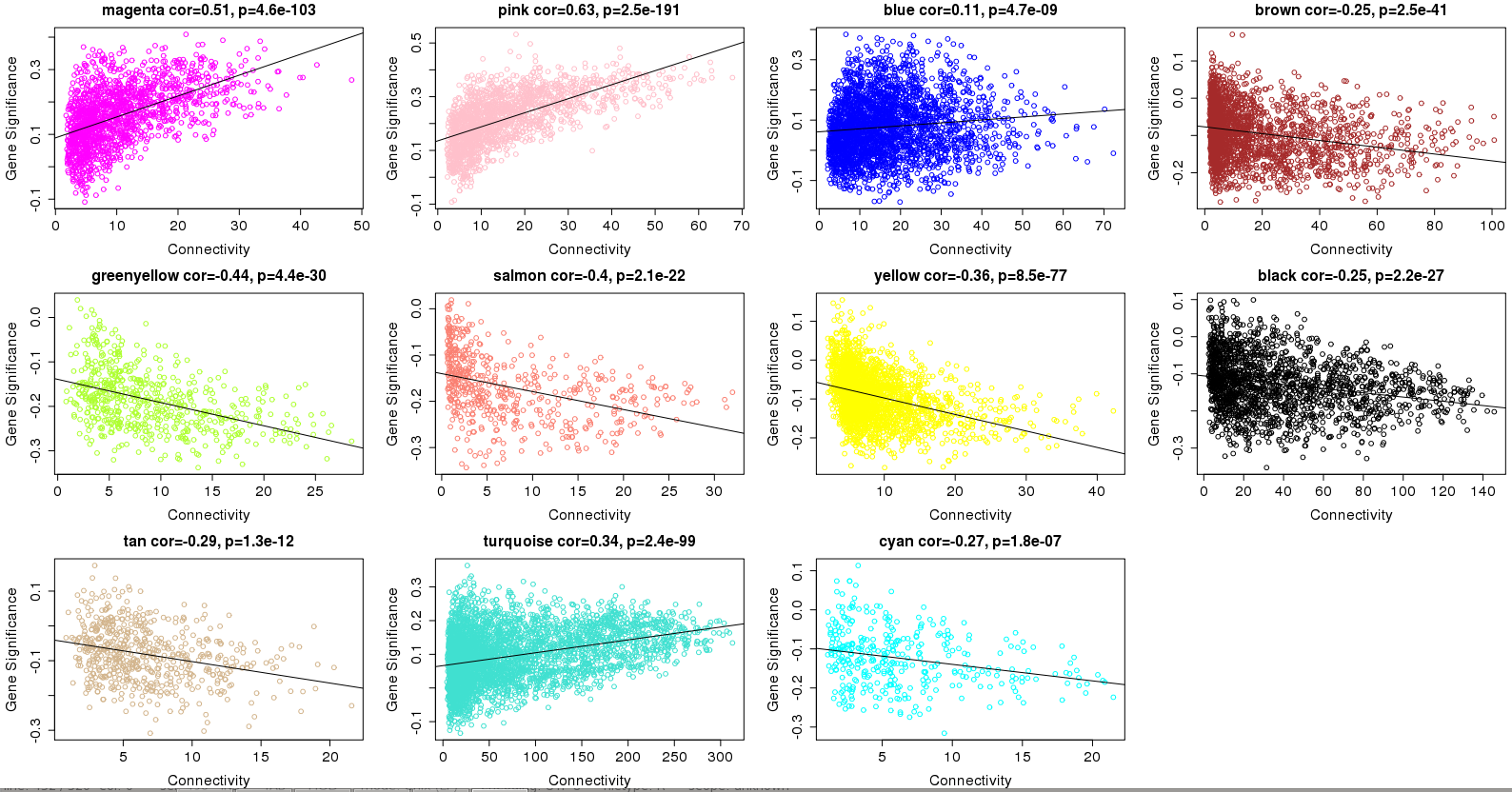


**Supplementary Information 8:** Correlations between Gene Significance (GS) and intramodular connectivity (*k*) for all modules of the biotic WGCNA network.
