# Supplementary figures and images for: The Use of PTI-Marker Genes to Identify Novel Compounds that Establish Induced Resistance in Rice
Source: Int J Mol Sci. 2020 Jan 2;21(1):317. doi: 10.3390/ijms21010317 (PMC6981679; doi:10.3390/ijms21010317)

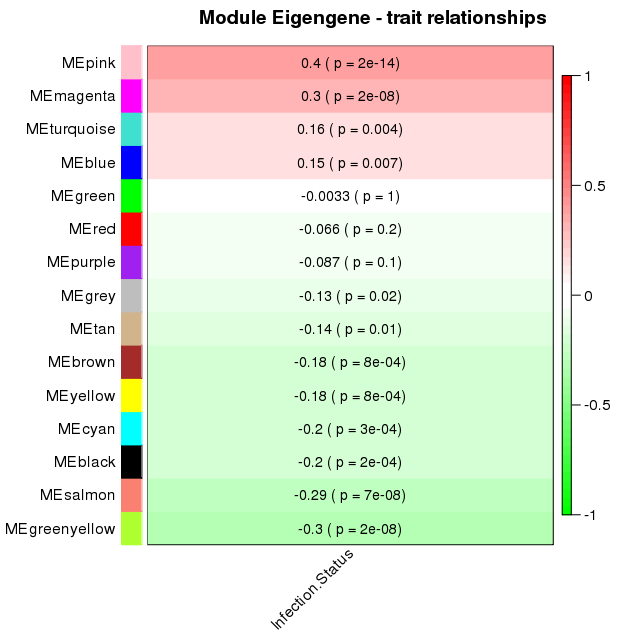

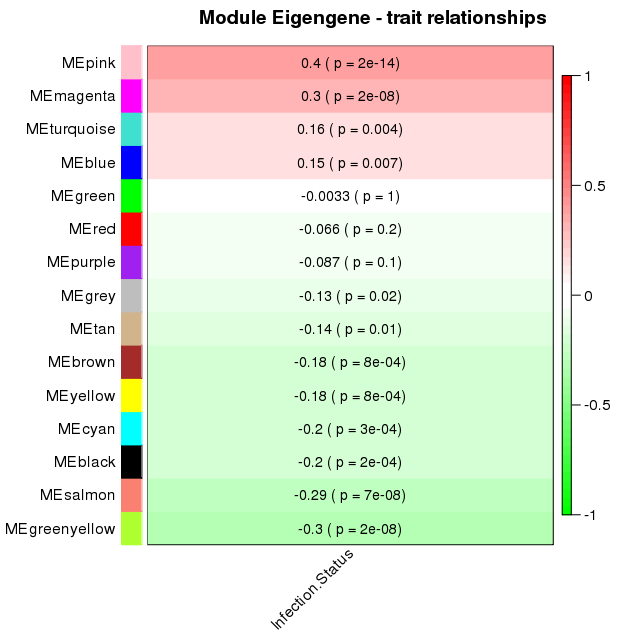


**Supplementary Information 4:** Module eigengenes (MEs) for all modules in the biotic WGCNA network.

Supplement: Supplementary file 1 [file ijms-21-00317-s001.zip › Supplementary_Information_4-MEs.docx]
